# Diversity of plant defense elicitor peptides within the Rosaceae

**DOI:** 10.1186/s12863-017-0593-4

**Published:** 2018-01-23

**Authors:** Cristina Ruiz, Anna Nadal, Laura Foix, Laura Montesinos, Emilio Montesinos, Maria Pla

**Affiliations:** Institute for Food and Agricultural Technology (INTEA), University of Girona, Campus Montilivi (EPS-1), 17003 Girona, Spain

**Keywords:** Plant elicitor peptide (pep), PROPEP, Plant defense, Rosaceae, *Prunus*, Pyreae, *Malus*, Ornamental, Pep diversity

## Abstract

**Background:**

Plant elicitor peptides (Peps) are endogenous molecules that induce and amplify the first line of inducible plant defense, known as pattern-triggered immunity, contributing to protect plants against attack by bacteria, fungi and herbivores. Pep topic application and transgenic expression have been found to enhance disease resistance in a small number of model plant-pathogen systems. The action of Peps relies on perception by specific receptors, so displaying a family-specific activity. Recently, the presence and activity of Peps within the Rosaceae has been demonstrated. Here we characterized the population of Pep sequences within the economically important plant family of Rosaceae, with special emphasis on the Amygdaleae and Pyreae tribes, which include the most relevant edible species such as apple, pear and peach, and numerous ornamental and wild species (e.g. photinia, firethorn and hawthorn).

**Results:**

The systematic experimental search for Pep and the corresponding precursor PROPEP sequences within 36 Amygdaleae and Pyreae species, and 100 cultivars had a highly homogeneous pattern, with two tribe-specific Pep types per plant, i.e. Pep1 and Pep2 (Amygdaleae) or Pep3 and Pep4 (Pyreae). Pep2 and Pep3 are highly conserved, reaching identity percentages similar to those of genes used in plant phylogenetic analyses, while Pep1 and Pep4 are somewhat more variable, with similar values to the corresponding PROPEPs. In contrast to Pep3 and Pep4, Pep1 and Pep2 sequences of different species paralleled their phylogenetic relationships, and putative ancestor sequences were identified. The large amount of sequences allowed refining of a C-terminal consensus sequence that would support the protective activity of Pep1–4 in a *Prunus* spp. and *Xanthomonas arboricola* pv. *pruni* system. Moreover, tribe-specific consensus sequences were deduced at the center and C-terminal regions of Peps, which might explain the higher protection efficiencies described upon topic treatments with Peps from the same tribe.

**Conclusions:**

The present study substantially enhances the knowledge on Peps within the Amygdaleae and Pyreae species. It can be the basis to design and fine-tune new control tools against important plant pathogens affecting *Prunus*, *Pyrus* and *Malus* species.

**Electronic supplementary material:**

The online version of this article (10.1186/s12863-017-0593-4) contains supplementary material, which is available to authorized users.

## Background

Plant immunity is triggered by the perception of elicitor molecules from pathogens or herbivores (pathogen- or herbivore-associated molecular patterns, PAMPs or HAMPs), or those originating endogenously within the host plant (damage-associated molecular patterns, DAMPs) [1]. Examples of well-characterized PAMPs are the bacterially derived peptide flg22 [2] and the fungal-derived chitin [3]. DAMPs include cutin monomers and cell wall fragments, such as oligogalacturonides or cellulose fragments, released upon infection [4, 5], and plant elicitor peptides (Peps) that are synthesized upon damage to trigger and amplify the innate immunity of plants to pathogens [6].

The plant immunity Pep and PEPR system structure and function has been extensively studied in the model plants *Arabidopsis* [7–12] and *Zea mays* [13, 14]. Peps are peptide sequences of 20–23 amino acids that mature from the carboxyl terminus of PROPEP precursor proteins [8]. They may be exported to the extracellular space or leak from disrupted cells [15, 16], and are recognized by transmembrane leucine-rich repeat kinase receptors (LRR-KRs) known as Pep receptors (PEPRs) of adjacent cells [9, 17]. Receptor activation results in production of reactive oxygen species (ROS), an increase in the plant hormones ethylene and jasmonic acid, and the accumulation of defense proteins and metabolites (reviewed in [15, 18]). It has been shown that exogenous application of Peps activates pattern-triggered immunity (PTI) [7], induces systemic immunity [19] and improves resistance to bacterial pathogens in the model plant *Arabidopsis* [9]. Pretreatment with Peps has also been found to protect maize against fungal infection [20] and herbivore attack [13]. Because the Pep/PEPR system activates multiple defense pathways, Huffaker and colleagues [15] hypothesized they might provide a strategy to increase plant resistance to pathogen attack in especially valuable crops.

Rosaceae species are important commercial plants extensively cultivated worldwide. The family includes trees producing pome fruits (apple and pear), stone fruits (cherry, peach, plum, nectarine, apricot, etc.) and nuts (almond), and also ornamental trees and shrubs (crabapple, flowering quince, cotoneaster, hawthorn, stranvaesia, etc.). Pome- and stone-fruits represent 22% of global fruit production [21]. The spread of transboundary plant pests in recent years can cause significant losses to farmers and threaten food security [22]. Economically important pathogens affecting Rosaceae species are *Erwinia amylovora* (fire blight), *Xanthomonas arboricola* pv. *pruni*, and *Pseudomonas syringae* pv. *persicae* (bacterial spot and canker of peach, prune, cherry and almond), all regulated as quarantine organisms by the EU Council directive 2000/29/EC [23] and by the European and Mediterranean Plant Protection Organization (EPPO) [24, 25]. Currently, control is based on eradication measures (e.g. destruction in situ of infected plants) and preventive treatment of the plants with chemical compounds (i.e. copper salts and antibiotics, the latter only permitted in some countries), with limited efficacy and a negative environmental impact.

Although PROPEP and PEPR orthologue genes are present in most angiosperms [14, 26], those from different plant families largely diverge at the amino acid level and family-specific Pep-motifs have been deduced. Coevolution of Peps and their receptors explains Pep intra-family compatibility and inter-family incompatibility [13, 14, 26], even though downstream pathways leading to PTI appear highly conserved. We recently identified two PROPEP and the corresponding mature Pep sequences in ten Rosaceae species and demonstrated that topic application of nanomolar doses enhanced resistance of *Prunus* spp. cultivars to challenge with the bacterial pathogen *Xanthomonas arboricola* pv. *pruni* [26]. There was compatibility within the Rosaceae Peps, but sequence variants could induce defense responses of different strength. In view of the economic importance of these species and the possible use of Peps to enhance plant resistance to a broad range of diseases, here we experimentally described the population of Pep sequences and their precursors over a wide representation of ornamental and edible varieties of the Rosaceae species with the highest commercial impact. This systematic approach led to the identification of 214 Pep sequences in 100 varieties from 36 Rosaceae species.

## Results

### Identification of Pep orthologues in the Rosaceae plant family

A selection of 100 Rosaceae commercial varieties, belonging to 36 species, was analyzed, including the most commercially relevant edible species within this family: apple, pear, peach and nectarine, plum, cherry, apricot, almond, quince and loquat, all belonging to the Pyreae and Amygdaleae tribes. To characterize these tribes we also analyzed a broad representation of ornamental genera that are largely commercialized in temperate regions. Among the Pyreae: bearberry cotoneaster (*Cotoneaster*); Callery pear (*Pyrus calleryana*); chokeberry (*Aronia*); crabapple (ornamental *Malus* species); flowering quince (*Chaenomeles*); firethorn (*Pyracantha*); hawthorn (*Crataegus*); medlar (*Mespilus*); stranvaesia (*Photinia*); service-tree (*Sorbus*) and shadbush (*Amelanchier*). Among the Amygdaleae: blackthorn (*P. spinosa*); cherry Accolade (*P.* ‘Accolade’); cherry laurel (*P. laurocerasus*); cherry plum (*P. cerasifera*); Chinese plum (*P. mume*); Fuji cherry (*P. incisa*); Higan cherry (*P.* x *subhirtella*) and Japanese cherry (*P. serrulata*). The final selection included 74 edible and 26 ornamental varieties (for the complete list see Tables 1 and 2).

**Table 1:**
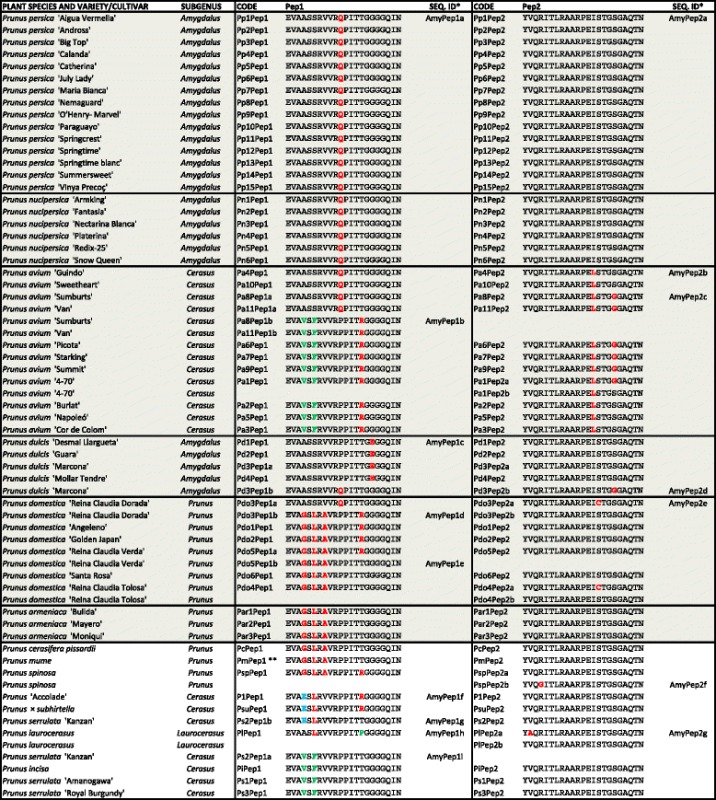
Plant elicitor peptide (Pep) amino acid sequences identified in 55 Amygdaleae varieties from 14 species

*SEQ. IDs are indicated in just one example per sequence

** [18]

Color codes indicate the frequency of a given amino acid at a given position: black corresponds to the most frequent amino acid and red, green and blue indicate decreasing frequencies. Peps from edible plant varieties are shaded in grey

**Table 2:**
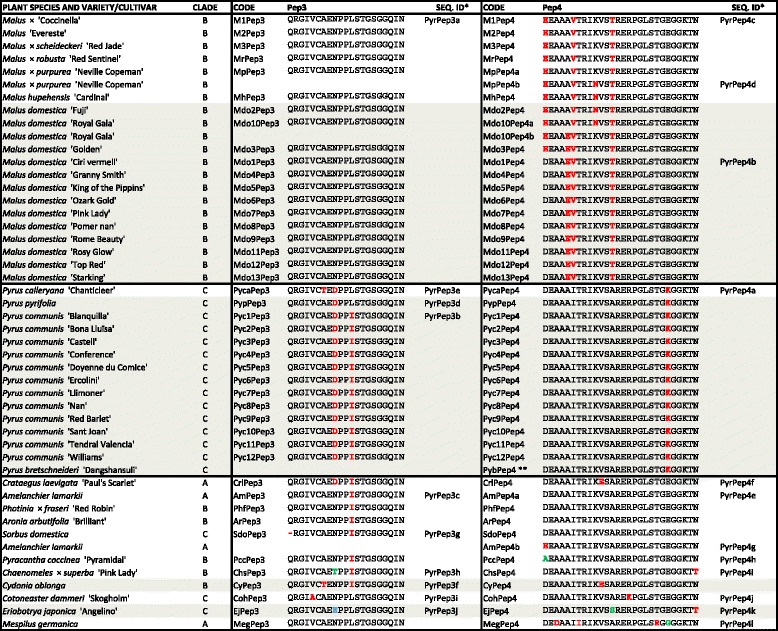
Plant elicitor peptide (Pep) amino acid sequences identified in 45 Pyreae varieties from 22 species

*SEQ. IDs are indicated in just one example per sequence

**Sequence published at GenBank

Color codes indicate the frequency of a given amino acid at a given position: black corresponds to the most frequent amino acid and red, green and blue indicate decreasing frequencies. Peps from edible plant varieties are shaded in grey

Pep sequences were PCR-amplified from leaf genomic DNA extracted from the different varieties using Pep1-, Pep2-, Pep3- and Pep4-specific primer pairs. These primer pairs were initially designed using, as target, sequences conserved among the small number of known Rosaceae *PROPEP* genes. To improve amplification of more distantly-related sequences, additional primer pairs were designed that targeted DNA regions highly conserved within the newly identified sequences. This approach resulted in the identification of two Pep sequences, Pep1 and Pep2, in each of the 55 Amygdaleae varieties analyzed (Table 1), and two distinct Pep sequences, Pep3 and Pep4, in all 45 Pyreae varieties (Table 2). This was in line with our previous identification of Pep1 and Pep2 in six Amygdaleae species and Pep3 and Pep4 in two Pyreae species [26]. No Pep1 or Pep2 sequences could be amplified in a selection of Pyreae species (*Malus domestica*, *Pyrus communis*, *Cotoneaster dammeri* and *Crataegus levigata*), and no Pep3 or Pep4 sequences were obtained on PCR analysis of representative Amygdaleae species (*P. persica*, *P. dulcis*, *P. domestica*, *P. avium*, *P. armeniaca*, *P. serrulata*, the specific varieties are given in Additional file 1). Similarly, Pep5, found in *Fragaria* species (which belong to the Potentilleae tribe, Rosaceae family), was not detected in any of these six Amygdaleae and four Pyreae representative species.

Most varieties had just one sequence for every Pep type (Pep1 and Pep2, or Pep3 and Pep4, according to the tribe). However, 18% of the Amygdaleae varieties (i.e. three *P. avium* and *P. domestica* varieties, and one of each, *P. dulcis*, *P. laurocerasus*, *P. serrulata* and *P. spinosa*), and 7% Pyreae varieties (*M. domestica* ‘Royal Gala’, *Malus x purpurea* and *Amelanchier lamarkii*) had two variants of the same Pep type.

The specific DNA and amino acid sequence of all identified Peps is shown in Additional file 1 (Pep1 and Pep2) and Additional file 2 (Pep3 and Pep4).

### Comparison of Rosaceae Peps

**Fig. 1:**
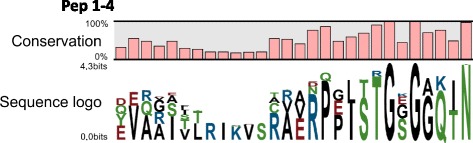
Identity comparison of plant elicitor peptides (Pep) amino acid sequences in 100 Rosaceae varieties. Depiction of the amino acid conservation and consensus sequences of aligned Pep sequences using the CLC tool

Comparison of up to 214 Rosaceae Pep sequences allowed a highly accurate description of the Pep motif in this family. Pep1–4 sequence conservation is illustrated in a sequence logo (Fig. 1). There were no major differences with the Rosaceae Pep-motif previously defined on the basis of 18 Pep sequences [26]. The highest conservation was at the level of the C-terminal amino acids, in agreement with previous reports [14, 26].

**Fig. 2:**
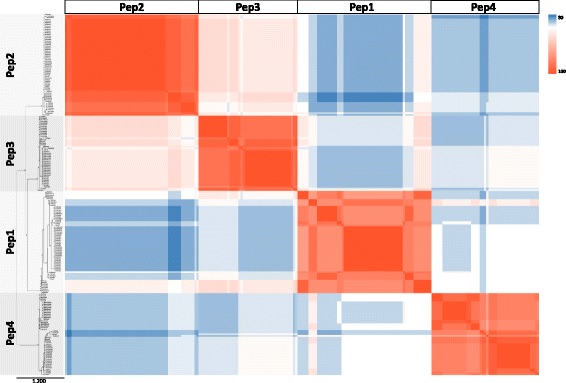
Identity comparison of plant elicitor peptides (Pep) amino acid sequences in 100 Rosaceae varieties. Heat map showing pairwise comparison of all 214 Pep sequences. Colors indicate increasing identity (%) from high (red), through white, to low (blue). The position of Pep1, Pep2, Pep3 and Pep4 sequences is indicated (top and left). Bootstrapped neighbor-joining tree of Pep amino acid sequences, constructed using the CLC tool (left). A higher-resolution image of the tree is displayed in Additional file 5

Overall, Pep1–4 amino acid sequences had pairwise identity values in the 30–100% range (Fig. 2) as assessed using the CLC alignment tool and the EMBL-EBI muscle algorithm. On neighbor-joining analysis, the Pep sequences clustered into four homology groups corresponding to Pep1, Pep2, Pep3 and Pep4 (Fig. 2). We thus independently aligned the Pep1, Pep2, Pep3 and Pep4 peptides identified in 55 varieties from 14 Amygdaleae species (Pep1 and Pep2) and 45 varieties from 22 Pyreae species (Pep3 and Pep4), and normalized pairwise identity values in order to obtain a single value per species to represent all varieties analyzed within the species. As shown in Table 3, Pep1, Pep2, Pep3 and Pep4 had different mean identity values (one-way ANOVA α < 0.05) in the 87% (Pep1) up to 98% (Pep2) range.

**Table 3:** Pep and N-terminal region of the precursor protein (Nt-PROPEP) amino acid sequence identity values

	homology groups				
	*a*	*b*	*c*	*d*	*e*
Pep1					86.8 ± 5.1
Pep2	98.1 ± 2.4				
Pep3		94.0 ± 4.0			
Pep4			90.0 ± 5.8	90.0 ± 5.8	
Nt-PROPEP1			90.6 ± 3.0	90.6 ± 3.0	
Nt-PROPEP2		94.0 ± 2.8			
Nt-PROPEP3				89.6 ± 7.3	
Nt-PROPEP4			91.6 ± 3.9		

Mean and standard deviations (SD) of normalized pairwise identity percentages of 61 Pep1, 61 Pep2, 44 Pep3 and 48 Pep4 peptides, and 50 Nt-PROPEP1, 56 Nt-PROPEP2, 41 Nt-PROPEP3 and 33 Nt-PROPEP4 are shown. Homology groups *a*-*e* correspond to statistically significant clusters obtained by one-way analysis of variance (ANOVA) and Tukey-b *post hoc* test with α < 0.05. Note that Pep1 and Pep2, and the corresponding PROPEPs, are only found in the Amygdaleae, and Pep3 and Pep4, and the corresponding PROPEPs, are uniquely found in the Pyreae

Separate comparison of the 61 Pep1, 61 Pep2, 44 Pep3 and 48 Pep4 sequences is shown in Tables 1 and 2, which depict all sequences and highlights all amino acid substitutions. Additional file 3 clearly shows the four derived consensus sequences as sequence logos. In agreement with their mean identity values, Pep2 and Pep3 were strikingly conserved within the analyzed Amygdaleae and Pyreae, respectively. Every Pep2 and Pep3 sequence had at most two amino acid substitutions when compared to the consensus, and in most cases they were either I^14^ > L^14^, S^18^ > G^18^ or both (Pep2), and N^9^ > D^9^, L^12^ > I^12^ or both (Pep3). In contrast, Pep1 and Pep4 had up to four-amino acid substitutions, which tended to be within the peptide N-terminal region. Remarkably, Pep1 and Pep4 both had at least one acidic amino acid at their N-terminal end, i.e. E^1^ in Pep1, and (D/E)^1^ E^2^ in Pep4.

Pep1–4 sequence variants had a tendency to cluster according to the plant species, even if there was sequence diversity within a given genus or species. As an example, there was complete conservation of Pep1 and Pep2 within *P. persica* and *P. nucipersica* varieties (a total of 21 varieties), and of Pep3 and Pep4 within *Pyrus communis* (12 analyzed varieties). *M. domestica* varieties shared the same Pep3 sequence but had some diversity in Pep4, while varieties from other species such as *P. avium* or *P. domestica* showed higher variability. In addition, certain sequence variants were spread throughout different plant groups e.g. *Sorbus domestica*, *Photinia*, *Aronia* and *Amelanchier* had the same Pep4 variant.

### Rosaceae Pep precursor sequences

Mature Peps derive from larger precursor PROPEP proteins. In *PROPEP* genes, the mature Pep and the remaining N-terminal portion (Nt-PROPEP) are encoded in two distinct exons. The strategy used to identify Peps from Rosaceae samples made it possible to sequence the complete *PROPEP* coding sequences: we obtained the sequences of 180 PROPEPs from 50 Amygdaleae and 45 Pyreae varieties. Their DNA and amino acid sequences are shown in Additional file 1 (PROPEP1 and PROPEP2) and Additional file 2 (PROPEP3 and PROPEP4).

**Fig. 3:**
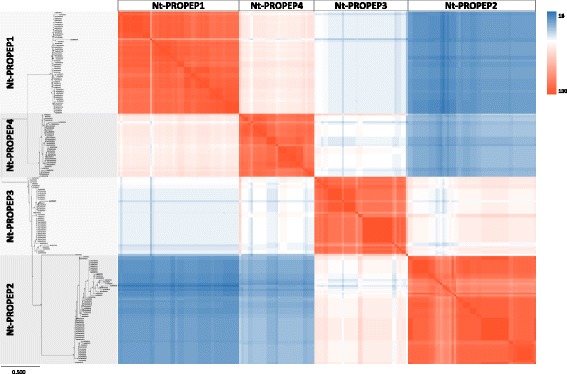
Identity comparison of the N-terminal portion of PROPEP amino acid sequences (i.e. omitting the Pep sequences) in 95 Rosaceae varieties. Heat map showing pairwise comparison of all 180 N-terminal PROPEP sequences. Colors indicate increasing identity (%) from high (red), through white, to low (blue). The position of N-terminal PROPEP1, PROPEP2, PROPEP3 and PROPEP4 sequences is indicated (top and left). Bootstrapped neighbor-joining tree of N-terminal PROPEP amino acid sequences, constructed using the CLC tool (left). A higher-resolution image of the tree is given in Additional file 6

Pairwise alignment of all Nt-PROPEP sequences showed a wide range of amino acid homologies i.e. from 100% down to 10%, as calculated with the CLC alignment and the EMBL-EBI muscle algorithms. As with the Pep sequences, neighbor-joining analysis of Nt-PROPEPs gave four clusters that corresponded to the N-terminal portions of PROPEP1, PROPEP2, PROPEP3 and PROPEP4 (Fig. 3). In contrast to Peps, the Nt-PROPEP1 and Nt-PROPEP4 were the most similar Nt-PROPEP types. Nt-PROPEP1, Nt-PROPEP2, Nt-PROPEP3 and Nt-PROPEP4 had mean identity percentages in the 89.6% ± 7.3 (Nt-PROPEP3) up to 94.0% ± 2.8 range (Nt-PROPEP2) as calculated using normalized Nt-PROPEP1–4 pairwise identity values (Table 3).

## Discussion

Plant elicitor peptides were first described as activating the PTI in *Arabidopsis* [7]. Current knowledge indicates that they are widely present within the angiosperms (for a review, see [18]). Up to now, a total of 92 Pep sequences from 42 angiosperm species have been reported, most belonging to Brassicaceae (33 Pep sequences in seven species), Poaceae (19 Peps in seven species), Rosaceae (18 Peps in nine species), Fabaceae (seven Peps in four species) and Solanaceae (six Peps in six species) species [14, 26]. Our aim was to extend the number of Pep sequences characterized within the Rosaceae family, experimentally searching for Peps in 36 different species and, for those with the highest economic impact, in up to 15 commercial varieties per species. Our systematic approach led to the identification of 214 Pep sequences in 100 Rosaceae varieties, resulting in this family being at the top of the list in terms of the number of reported sequences.

Here we experimentally demonstrated that plants belonging to the same tribe have similar Peps: all analyzed Amygdaleae species and varieties had both, and only, Pep1 and Pep2, while the Pyreae had both, and only, Pep3 and Pep4 sequences. This is in agreement with previous observations with just seven species from these tribes. In general, plants seem to have one to three Peps, with the remarkable exceptions of *Arabidopsis thaliana*, with Pep1–8, [10], and other Brassicaceae species and *Zea mays*, with four to six Peps, [13, 14]. The systematic search for Peps in a wide range of the most relevant Rosaceae tribes consistently showed the presence of two Peps per species or variety. Although the presence of additional dissimilar Pep sequences cannot be fully discarded, this homogeneity has not been described in other plant families to date.

The Amygdaleae and the Pyreae tribes include numerous species that are cultivated worldwide. As with most important crops, different varieties of each species, with specific traits, are commercialized: these may be native varieties or those obtained with specific features such as fruit characteristics, agronomic performance, flower appearance, foliage, vigor, or tolerance to abiotic and biotic stress. Within the background of this phenotypic and genetic diversity, pairwise alignment of Pep sequences from up to 55 Amygdaleae and 45 Pyreae samples showed that Pep2 and Pep3 had amino acid identity values of 98 and 94%, respectively, while Pep4 and Pep1 were slightly less conserved (90 and 87%, respectively). These identity values were compared to those for highly conserved sequences typically used in plant phylogenetic analyses such as the chloroplast ribulose-bis-phosphate carboxylase large subunit (RbcL) [27]. In an *in silico* exercise, aligning up to 1100 Amygdaleae and 750 Pyreae RbcL sequences available at GenBank, we found amino acid identity values of 98.8% ± 1.0 and 99.2% ± 0.8, respectively. Besides those used to establish phylogenetic relationships between species, gene sequences are normally available in a small number of Rosaceae species. As an example of defence related genes, on alignment of 11 accessible NPR3 sequences (nonexpresser of pathogenesis-related genes 3, Moreau et al., 2012), these values were lower (81.7% ± 17.3 and 82.1% ± 18.6 in the Amygdaleae and Pyreae, respectively). In line with these values, Pep2 and, to a somewhat lower extent Pep3, can be considered highly conserved peptides.

The present report adds up to 180 PROPEP1–4 sequences from 34 Rosaceae species and 95 varieties. As indicated, Peps are synthesized as larger PROPEP sequences. While Peps have a recognized role in modulation of plant defenses against pathogens [6, 15, 18], expression analyses and subcellular localization of the eight *Arabidopsis thaliana* PROPEPs (PROPEP1–8) suggest possible additional roles of the precursor proteins in plant development and reproduction [10]. Even though they are encoded in a single gene, the N-terminal portion of PROPEP2 and PROPEP3 (i.e. the PROPEP sequences except for the mature Peps) were less conserved than the corresponding Peps. This phenomenon was exclusive to the well-conserved Pep2 and Pep3 in the Amygdaleae and the Pyreae, respectively. Conversely, the N-terminal portion of (Amygdaleae) PROPEP1 and (Pyreae) PROPEP4, generated after maturation of the less conserved Pep1 and Pep4, respectively, had lower or similar identity percentages compared to the corresponding Peps. The greater preservation of mature Pep2 and Pep3 sequences seems to indicate that they play an important role in the Amygdaleae and Pyreae, respectively, which depends on their precise sequence. Peps activate and modulate defense responses through specific interaction with the LRR domain of Pep receptors. Through crystal structure of the *A. thaliana* PEPR1LRR-AtPep1, Tang and colleagues [28] suggested that PEPR1 recognition of the C-terminal amino acid motif of AtPep1 determines the specific interaction. In particular, S^15^, G^17^ and N^23^ were critical for binding [28, 29]. Of these, G and N were fully conserved within the Rosaceae while S was very occasionally substituted by a similar hydroxylic amino acid, suggesting they may be fundamental to Pep and PEPR interaction also in this plant family. Nevertheless, the N-terminal portion of Peps, known to be more variable, has also been shown to have a role in modulating signal transduction and may have an effect on the extent of the defense response [28]. The unusual conservation of Pep2 and Pep3 suggests that the precise sequence, in its entirety, drives optimal interaction with the Amygdaleae and Pyreae receptors, respectively, and transduction of the defense signal. Similar to the PROPEP1–4 N-terminal sequences, Pep1 and Pep4 are more prone to amino acid changes. The measured variability of Pep sequences might hypothetically be linked to the necessary interaction with receptor and co-receptor molecules (such as BRI1-associated kinase 1 [BAK1] [30]) and, at the same time, to the evolving nature of microbial infection mechanisms.

**Fig. 4:**
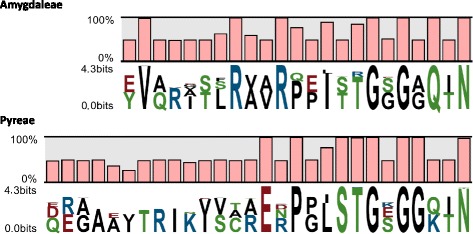
Comparison of the amino acid sequences of aligned plant elicitor peptides (Pep) in 55 Amygdaleae (Pep1 and Pep2) and 45 Pyreae (Pep3 and Pep4) varieties. Sequence logos are represented for every tribe. Bars indicate sequence conservation at every position

There is a recognized compatibility within Peps from the same plant family in eliciting PTI-like responses, due to sharing the same Pep C-terminal motif [8, 14]. We have recently described the Rosaceae consensus sequence on the basis of 18 Peps from six Amygdaleae, two Pyreae and two Potentilleae species [26]. Here, the Rosaceae Pep motif could be confirmed because of the substantial increase in the number of Pep sequences and analyzed species, with special emphasis on the economically relevant Amygdaleae and Pyreae tribes. Nevertheless, these two tribes have different Pep types and the Rosaceae PEPR-LRR binding domains have a parallel clustering pattern [26], which can be understood as an example of the coevolution of these molecules to optimize Pep-mediated defense responses [14]. In addition to the Rosaceae C-terminal motif, there were a few amino acid positions at the C-terminal and central portions of Peps that were also fully conserved within every tribe (Fig. 4). This might explain why Pep3 and Pep4 (Pyreae) did not increase the resistance of *Prunus* spp. (Amygdaleae) leaves to infection with the bacterial pathogen *Xanthomonas arboricola* pv. *pruni,* with the same level of efficiency as Pep1 and Pep2 (from the same Amygdaleae tribe) [26]. Amino acids characteristic of a tribe might be involved in enhancement or fine-tuning of Pep and PEPR-LRR binding.

**Fig. 5:**
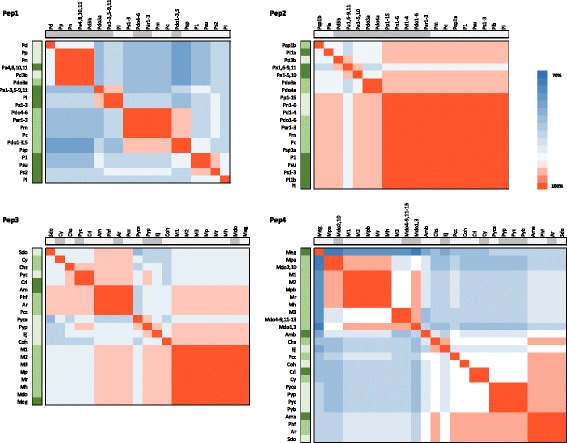
Comparison of the amino acid sequences of aligned plant elicitor peptides (Pep) Pep1, Pep2, Pep3 and Pep4 in 14 Amygdaleae and 22 Pyreae species. The heat map shows pairwise identity values. Note that, the same sequence variant found in different varieties from the same species, is shown in a single row and column. Colors indicate increasing identity from high (red), through white, to low (blue). The position of edible and ornamental species is indicated at the top of every panel (dark grey, edible; light gray, ornamental). The position of the different phylogenetic groups is shown at the left: Amygdaleae, from dark to light green, *Cerasus*, *Prunus* and *Amygdalus*; Pyreae, from dark to light green, clades A, B and C. Am, *Amelanchier lamarkii*; Ar, *Aronia arbutifolia*; Chs, *Chaenomeles* x *superba*; Coh, *Cotoneaster dommeri*; Crl, *Crataegus laevigota*; Cy, *Cydonia* spp.; Ej, *Eriobotrya japonica*; M1, *Malus* x ‘Coccinella’; M2, *Malus* ‘Evereste’; M3, *Malus* x *scheideckeri*; Mdo, *Malus domestica*; Meg, *Mespilus germanica*; Mh, *M. hupehensis*; Mp, *Malus* x *purpurea*; Mr., *Malus* x *robusta*; P1, *Prunus* ‘Accolade’; Pa, *Prunus avium*; Par, *P. armeniaca*; Pc, *P. cerasifera*; Pcc, *Pyracantha coccinea*; Pd, *P. dulcis*; Pdo, *P. domestica*; Phf, *Photinia* x *fraseri*; Pi, *P. incisa*; Pl, *P. laurocerasus*; Pm, *P. mume*; Pn, *P. nucipersica*; Pp, *P. persica*; Ps, *P. serrulata*; Psp, *P. spinosa*; Psu, *Prunus* x *subhirtella*; Pyb, *Pyrus bretschneideri*; Pyc, *Pyrus communis*; Pyca, *Pyrus calleryana*; Pyp, *Pyrus pyrifolia*; and Sdo, *Sorbus domestica*

The taxonomy of the Amygdaleae tribe has been controversial. It includes the genus *Prunus*, which, historically, has been divided into five subgenera: *Amygdalus*, *Cerasus*, *Laurocerasus*, *Padus* and *Prunus* [31, 32]. Phylogenetic clustering based on the ribosomal DNA internal transcribed spacer (ITS) sequences [33], either combined or not with the nuclear gene sorbitol-6-phosphate dehydrogenase (*s6pdh*) and the chloroplast *trnL-trnF* spacer [34], gave two major groups within *Prunus* that corresponded to *Amygdalus-Prunus* and *Cerasus–Laurocerasus–Padus* subgenera. Here we analyzed up to 14 *Prunus* species that belonged to the *Amygdalus* (Pd, Pn, Pp), *Prunus* (Par, Pc, Pdo, Pm, Psp) and *Cerasus–Laurocerasus* (P1, Pa, Pi, Pl, Ps, Psu) subgenera. Close analysis of the Pep sequence variants demonstrated a correlation between the Pep1 and Pep2 sequences in the different species and their phylogenetic links (Fig. 5). Remarkably, the major form of Pep2 (YVQRITLRAARPEISTGSGAQTN, AmyPep2a) appeared in all analyzed subgenera and in all species except *P. avium*. Nevertheless, the most common *P. avium* Pep2 variant has a single conservative I^14^ > L^14^ amino acid substitution compared to AmyPep2a. This strongly suggests that AmyPep2a was present in the common ancestor within the Amygdaleae and peptide diversification occurred from this variant. Similarly, there was one Pep1 variant (EVAASSRVVRQPITTGGGGQIN, AmyPep1a) common to most *Amygdalus,* one *Prunus* and one *Cerasus-Laurocerasus* species, suggesting it might correspond to the ancestral sequence. All other Pep1 and Pep2 variants were found in just one subgenus, indicating their appearance after phylogenetic branching. *P. persica* and *P. nucipersica* varieties had exclusively the ancestral Amygdaleae AmyPep1a and AmyPep2a variants. Other species, such as *P. avium*, had higher sequence diversity: the specific combination of Pep1 and Pep2 variants found in any given commercial variety reflects the corresponding breeding crosses. Finally, some varieties simultaneously had two variants of Pep1 and/or Pep2, occurring mainly, as it might be expected, in polyploid species such as *P. domestica* (tetraploid), *P. laurocerasus* and *P. spinosa* (hexaploid) [35]. When this occurred, the two coexisting peptides were also found in other varieties from the same species or even in different species, reflecting the crossings to obtain every variety. As an example, *P. avium* ‘Van’ had two Pep1 variants: the probable ancestor AmyPep1a, also found in varieties such as *P. avium* ‘Sweetheart’ and in species such as *P. persica*, and AmyPep1b, only common to *P. avium* varieties such as ‘Starking’. The putative ancestral AmyPep2a variant coexisted with a new unique variant in three examples (V^2^ > R^2^ in *P. laurocerasus*, R^4^ > G^4^ in *P. spinosa* and S^18^ > G^18^ in *P. avium* ‘Marcona’), either indicating that a change occurred after divergence of this particular species or reflecting crossings with other species not included in our analysis.

A number of publications describe 26 genera within the Pyreae on the basis of morphological, anatomical and phytochemical data [36, 37], and, on analysis of combined chloroplast and nuclear ITS sequence data, they were grouped into three major clades (above the early diverging *Kaganeckia*, *Lindleya* and *Vauquilinia* lineages) [38]. Here we identified Pep3 and Pep4 sequences from half recognized Pyreae genera representative of all three major clades: *Amelanchier*, *Mespillus* and *Crataegus* (clade A), *Photinia*, *Pyracantha*, *Cydonia*, *Aronia*, *Chaenomeles* and *Malus* (clade B), and *Eriobotrya*, *Cotoneaster*, *Pyrus* and *Sorbus* (clade C). As assessed by pairwise identity comparison, Pep3 and Pep4 variants in our samples did not cluster according to the plant clades, and no clear ancestral variants could be identified (Fig. 5). Instead, various peptide forms were shared by plant genera belonging to different clades, which might be linked to the weak reproductive barriers known to exist between different Pyreae species and leading to hybridization.

Commercial Rosaceae species are both edible and ornamental, and are intermingled with each other in phylogenetic trees [33, 34, 38]. However, while some ornamental varieties are commercialized in their native forms, breeding of interesting edible and ornamental varieties obeys visibly different criteria. Interestingly, Pep1, Pep2, Pep3 and Pep4 sequences from edible and ornamental species did not cluster into separate groups, which seems to suggest that Pep variants were not linked to the selection criteria associated to either edible or ornamental varieties.

## Conclusion

Peps have been reported to improve the resistance of various plant species to pathogen infection both through overexpression and external application [7, 9, 13, 20, 26]. Here we substantially increased the knowledge on these defense molecules through extensive sequencing and comparison of Pep sequences from the majority of Pyreae and Amygdaleae species and a selection of commercial varieties. There was a clear pattern of two Pep types in every plant species, which are tribe specific and have conservation rates within the 87 to 98% range. The present characterization of Rosaceae Peps can sustain optimization of new tools to control pathogens in economically relevant edible species such as apple, pear and peach, as well as in numerous ornamental trees and shrubs. We propose tribe consensus sequences as the basis to design more efficient and specific protection of Rosaceae cultures belonging to different tribes, and work is in progress to further explore this possibility.

## Methods

### Plant materials

Leaves from the commercial Rosaceae species listed in Table 1 were obtained from professional growers (Soljardí S.L., Jafre, Spain; Nou Jardí, Banyoles, Spain; Tortadès, Sant Hilari Sacalm, Spain), thoroughly washed with deionized water and used for DNA extraction. All plant materials were accompanied with the corresponding label and phytosanitary passport according to European rules.

### Nucleic acids extraction, PCR amplification and sequencing

Genomic DNA from 100 mg plant leaves was extracted using the commercial NucleoSpin^R^ Plant II kit (Macherey-Nagel, Düren, Germany) according to the manufacturer’s instructions. It was quantified by UV absorption at 260 nm in a NanoDrop ND1000 spectrophotometer (Nanodrop technologies, Wilmington, DE), and the OD 260/280 and 260/230 nm absorption ratios used to confirm the purity of the DNA samples.

As a general approach, PCR amplification of PROPEP and Pep sequences was carried out using specific primers designed to target conserved sequences in the 5′ and 3′ noncoding regions of the PROPEP sequences available in silico [26]. In case of lack of amplification, PCR annealing temperature was decreased and, when needed, additional primers were designed based on alignment including the newly obtained sequences. For every species and variety PROPEP1, PROPEP2, PROPEP3 and PROPEP4 sequences were separately amplified. Specifically, the PROPEP and Pep sequences of Amygdaleae varieties were PCR-amplified using PROPEP1- and PROPEP2- specific primer pairs [26] designed based on the only four sequences available in silico, i.e. *P. mume* and *P. persica* PROPEP genes (NC_024131.1 regions 16,562,542–16,563,168 and 16,566,785–16,567,312, and NC_034016.1 regions 5,274,001–5,274,638 and 5,299,005–5,299,443). There was specific amplification of PROPEP1 and PROPEP2 from all studied species and varieties except for two *P. avium* varieties’ PROPEP1 (‘cor colom’ and ‘Napoleó’); and an additional reverse primer was designed taking into account the 46 newly obtained PROPEP1 sequences (Additional file 1). Similarly, the PROPEP and Pep sequences of Pyreae varieties were amplified with primers targeting the 5′ and 3′ noncoding regions of PROPEP3 and PROPEP4. They were designed on the basis of the only four sequences described to date, i.e. *Malus domestica* var. ‘Golden Delicious’ and *Pyrus x bretschneideri* var. ‘Dangshansuli’ PROPEP genes (NW_007545668.1 region 1,666,343–1,666,648, NC_024247.1 region 22,849,040–22,849,667, NW_008988545.1 region 11,359–11,968 and NW_008988574.1 region 173,072–173,672). On analysis of genomic DNA extracted from ten species and varieties there was no PROPEP3 positive amplification, and an additional primer pair was designed based on the 31 newly obtained sequences (Additional file 2). Initial PROPEP4 analyses produced 25 new sequences (out of 44 species and varieties), which were used to design an additional PROPEP4 primer pair and obtain 8 extra sequences. Furthermore, all 33 sequences were aligned to design a third set of primers, which permitted amplifying PROPEP4 from 10 varieties (Additional file 2). All PROPEP primers, specifying their use for the different species and varieties, are shown in Additional file 4. Finally, a new primer pair was designed targeting 5′ and 3′ noncoding regions of PROPEP5, based on the available sequences i.e. *Fragaria ananassa* FaPROPEP5a and FaPROPEP5b and *F. vesca* FvPROPEP5 [26] (BATT01285995.1, BATT01119275.1 and NC_020496.1 region 2,820,573–2,821,588). A selection of four Pyreae and six Amygdaleae species (Additional file 4), representing these tribes, were tested with all primer pairs to evaluate the specificity of every PROPEP type.

PCR assays were carried out as described [26]. The final volume was 50 μL in 1× reaction buffer with 1.5 mM Mg^2+^ and 300 nM each primer (Sigma, Mannheim, Germany), 200 μM dNTPs and 2.5 U/μl unit Expand High Fidelity DNA polymerase (Roche Diagnostics Corporation, GmbH, Mannheim, Germany). The reaction conditions were as follows: 2 min at 94 °C; 10 cycles of 15 s at 94 °C, 30 s at the appropriate annealing temperature (Additional file 4) and 1 min at 72 °C; 20 cycles of 15 s at 94 °C, 30 s at the same annealing temperature and 1 min, plus an additional 5 s for each successive cycle, at 72 °C; and a final extension of 7 min at 72 °C.

PCR products were purified using the NucleoSpin Plant II Kit (Macherey-Nagel, Barcelona, Spain) and sequenced (Macrogen Europa, Amsterdam, The Netherlands). This approach led to clear sequences. In some cases, where ambiguities at specific nucleotide positions were found, the PCR products were cloned in the pSpark vector (pSpark DNA cloning system, Canvax, Córdoba, Spain) and five positive clones were sequenced in order to identify the possible presence of more than one sequence in the same genome.

### Bioinformatics

We used the GeneMark tool [39] for intron prediction and ExPASy [40] for sequence translation. CLC Main workbench 6.9.1 (CLC bio, Aarhus, Denmark) was used for protein alignment and building of identity graphs and phylogenetic trees through neighbour-joining with Kimura protein distance measure and 1000 bootstraps. The same software was used to construct sequence logos to visualize Pep consensus sequences.

The EMBL-EBI muscle tool was used for sequence pairwise comparison and drawing of identity heat maps.

## Additional files

Additional file 1: DNA and protein sequences of the identified Amygdaleae Pep and, in many examples, PROPEP sequences. Intron sequences are shown in lower case. In specific cases a given variety contained two sequences: the different nucleotide and amino acid positions are indicated with a bar. (XLSX 45 kb)

Additional file 2: DNA and protein sequences of the identified Pyreae Pep and, in many examples, PROPEP sequences. Intron sequences are shown in lower case. In specific cases a given variety contained two sequences: the different nucleotide and amino acid positions are indicated with a bar. (XLSX 27 kb)

Additional file 3: Depiction of the consensus sequences of aligned Pep1, Pep2, Pep3 and Pep4 sequences. Sequence logos are represented for every Pep. Bars indicate sequence conservation at every position. (PDF 282 kb)

Additional file 4: Primers used in this work, with their use and optimal reaction conditions. (XLSX 13 kb)

Additional file 5: Bootstrapped neighbor-joining tree of 214 Rosaceae plant elicitor peptide (Pep) amino acid sequences, constructed using the CLC tool. (PDF 1227 kb)

Additional file 6: Bootstrapped neighbor-joining tree of 180 N-terminal regions of PROPEP amino acid sequences from 95 Rosaceae varieties, constructed using the CLC tool. (PDF 1327 kb)

## Abbreviations

LRR: Leucine rich repeat domain
Pep: Plant elicitor peptide
PEPR: Pep receptor
PROPEP: Pep precursor

## References

[1] Boller T, Felix G (2009). A renaissance of elicitors: perception of microbe-associated molecular patterns and danger signals by pattern-recognition receptors. Annu Rev Plant Biol.

[2] Zipfel C, Robatzek S, Navarro L, Oakeley EJ, Jones JDG, Felix G (2004). Bacterial disease resistance in Arabidopsis through flagellin perception. Nature.

[3] Lizasa EI, Mitsutomi M, Nagano Y (2010). Direct binding of a plant LysM receptor-like kinase, LysM RLK1/CERK1, to chitin in vitro. J Biol Chem.

[4] Ferrari S, Savatin DV, Sicilia F, Gramegna G, Cervone F, De Lorenzo G. Oligogalacturonides: plant damage-associated molecular patterns and regulators of growth and development. Front Plant Sci. 2013;4(49) 10.3389/fpls.2013.00049. 10.3389/fpls.2013.00049 PMC3595604 23493833

[5] Nühse TS (2012). Cell wall integrity signaling and innate immunity in plants. Front Plant Sci.

[6] Albert M (2013). Peptides as triggers of plant defence. J Exp Bot.

[7] Huffaker A, Pearce G, Ryan CA (2006). An endogenous peptide signal in Arabidopsis activates components of the innate immune response. Proc Natl Acad Sci U S A.

[8] Huffaker A, Ryan CA (2007). Endogenous peptide defense signals in Arabidopsis differentially amplify signaling for the innate immune response. Proc Natl Acad Sci U S A.

[9] Yamaguchi Y, Huffaker A, Bryan AC, Tax FE, Ryan CA (2010). PEPR2 is a second receptor for the Pep1 and Pep2 peptides and contributes to defense responses in Arabidopsis. Plant Cell.

[10] Bartels S, Lori M, MBengue M, Verk M, Klauser D, Hander T (2013). The family of peps and their precursors in arabidopsis: differential expression and localization but similar induction of pattern-triggered immune responses. J Exp Bot.

[11] Tintor N, Ross A, Kanehara K, Yamada K, Fan L, Kemmerling B (2013). Layered pattern receptor signaling via ethylene and endogenous elicitor peptides during Arabidopsis immunity to bacterial infection. Proc Natl Acad Sci U S A.

[12] Klauser D, Desurmont GA, Glauser G, Vallat A, Flury P, Boller T (2015). The Arabidopsis pep-PEPR system is induced by herbivore feeding and contributes to JA-mediated plant defence against herbivory. J Exp Bot.

[13] Huffaker A, Pearce G, Veyrat N, Erb M, Turlings TCJ, Sartor R (2013). Plant elicitor peptides are conserved signals regulating direct and indirect antiherbivore defense. Proc Natl Acad Sci U S A.

[14] Lori M, Van Verk MC, Hander T, Schatowitz H, Klauser D, Flury P (2015). Evolutionary divergence of the plant elicitor peptides (peps) and their receptors: interfamily incompatibility of perception but compatibility of downstream signalling. J Exp Bot.

[15] Yamaguchi Y, Huffaker A (2011). Endogenous peptide elicitors in higher plants. Curr Opin Plant Biol.

[16] Ding B, Chen Z (2012). Molecular interactions between cell penetrating peptide Pep-1 and model cell membranes. J Phys Chem B.

[17] Krol E, Mentzel T, Chinchilla D, Boller T, Felix G, Kemmerling B (2010). Perception of the Arabidopsis danger signal peptide 1 involves the pattern recognition receptor AtPEPR1 and its close homologue AtPEPR2. J Biol Chem.

[18] Bartels S, Boller T (2015). Quo vadis, pep? Plant elicitor peptides at the crossroads of immunity, stress, and development. J Exp Bot.

[19] Ross A, Yamada K, Hiruma K, Yamashita-Yamada M, Lu X, Takano Y (2014). The Arabidopsis PEPR pathway couples local and systemic plant immunity. EMBO J.

[20] Huffaker A, Dafoe NJ, Schmelz EA (2011). ZmPep1, an ortholog of Arabidopsis elicitor peptide 1, regulates maize innate immunity and enhances disease resistance. Plant Physiol.

[21] Food and Agriculture Organization of the United Nations. FAOSTAT statistics. Database. 2017; http://www.fao.org/faostat/en/#home. Accessed 10 Mar 2017

[22] Food and Agriculture Organization of United Nations. http://www.fao.org/home/en/ (2017). Accessed 20 Mar 2017.

[23] European Union (EU) Council Directive 2000/29/EC of 8 May 2000 on protective measures against the introduction into the Community of organisms harmful to plants or plant products and against their spread within the Community. Off J Eur Communities. 2000;L169:1–112.

[24] EPPO/OEPP. Data sheets on quarantine organisms *Xanthomonas arboricola* pv. *pruni*. 2003. https://www.eppo.int/QUARANTINE/data_sheets/bacteria/XANTPR_ds.pdf. Accessed 6 Feb 2017.

[25] EPPO/OEPP. PQR - EPPO database on quarantine pests. 2015. https://www.eppo.int/DATABASES/pqr/pqr.htm. Accessed 6 Feb 2017.

[26] Ruiz C, Nadal A, Montesinos M, Pla M. Novel Rosaceae plant elicitor peptides as sustainable tools to control Xanthomonas arboricola pv. Pruni in Prunus spp. Mol Plant Pathol. 2017; 10.1111/mpp.12534. 10.1111/mpp.12534 PMC6638028 28056495

[27] Savolainen V, Chase MW, Hoot SB, Morton CM, Soltis DE, Bayer C (2000). Phylogenetics of flowering plants based on combined analysis of plastid atpB and rbcL gene sequences. Syst Biol.

[28] Tang J, Han Z, Sun Y, Zhang H, Gong X, Chai J (2015). Structural basis for recognition of an endogenous peptide by the plant receptor kinase PEPR1. Cell Res.

[29] Roux M, Schwessinger B, Albrecht C, Chinchilla D, Jones A, Holton N (2011). The arabidopsis leucine-rich repeat receptor-like kinases BAK1/SERK3 and BKK1/SERK4 are required for innate immunity to Hemibiotrophic and biotrophic pathogens. Plant Cell.

[30] Schulze B, Mentzel T, Jehle AK, Mueller K, Beeler S, Boller T (2010). Rapid heteromerization and phosphorylation of ligand-activated plant transmembrane receptors and their associated kinase BAK1. J Biol Chem.

[31] Rehder A. Manual of cultivated trees and shrubs. Hardy in North America exclusive of the subtropical and warmer temperate regions. 2nd ed. New York: The Macmillan Company; 1940.

[32] Mowrey BD, Werner DJ (1990). Phylogenetic relationships among species of Prunus as inferred by isozyme markers. TAG Theor Appl Genet.

[33] Lee S, Wen J (2001). A phylogenetic analysis of Prunus and the Amygdaloideae (Rosaceae) using ITS sequences of nuclear ribosomal DNA. Am J Bot.

[34] Bortiri E, SH O, Gao FY, Potter D (2002). The phylogenetic utility of nucleotide sequences of sorbitol 6-phosphate dehydrogenase in Prunus (Rosaceae). Am J Bot.

[35] Webster AD. The origin, distribution and genetic diversity of temperate tree fruits. In: Tromp J, Webster AD, Wertheim SJ, editors. Fundam Temp Zo tree fruit Prod. Leiden, the Netherlands: Backhuys Publishers; 2005. p. 1–11.

[36] Kovanda M. On the generic limits in the Maloideae. 1965;37:27–34.

[37] Kalkman C, Kubitzki K (2004). Rosaceae. Flower plants – Dicotyledons Celastrales, Oxalidales, Rosales, Cornales, Ericales.

[38] Lo EYY, Donoghue MJ (2012). Expanded phylogenetic and dating analyses of the apples and their relatives (Pyreae, Rosaceae). Mol Phylogenet Evol.

[39] Besemer J, Borodovsky M (2005). GeneMark: web software for gene finding in prokaryotes, eukaryotes and viruses. Nucleic Acids Res.

[40] Gasteiger E, Gattiker A, Hoogland C, Ivanyi I, Ron DA, Bairoch A (2003). ExPASy: the proteomics server for in-depth protein knowledge and analysis. Nucleic Acids Res.

