# Supplementary figures and images for: Diversity of plant defense elicitor peptides within the Rosaceae
Source: BMC Genet. 2018 Jan 23;19:11. doi: 10.1186/s12863-017-0593-4 (PMC5782389; doi:10.1186/s12863-017-0593-4)

Additional file 3

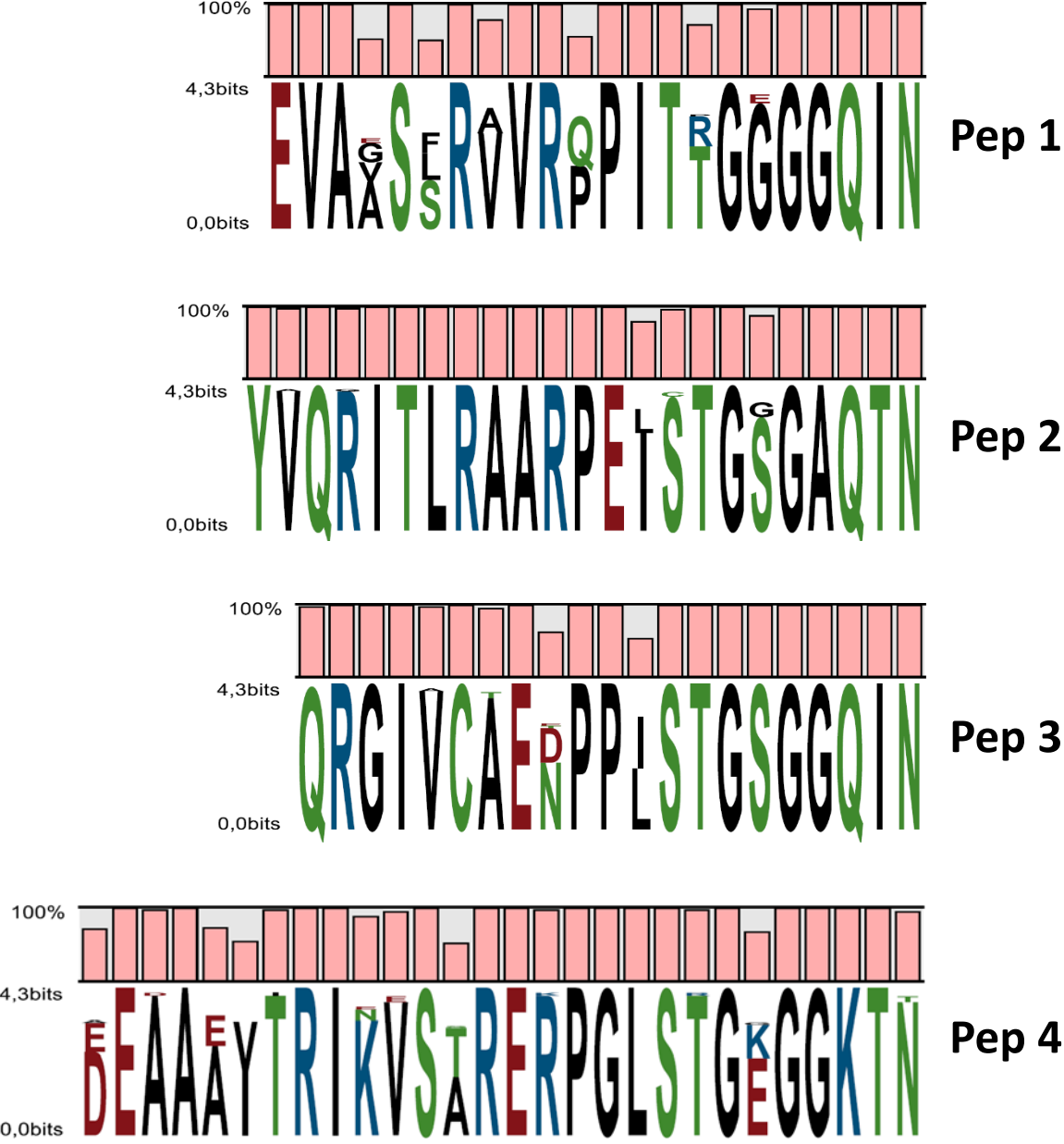

Supplement: Supplementary file 3 — Depiction of the consensus sequences of aligned Pep1, Pep2, Pep3 and Pep4 sequences. Sequence logos are represented for every Pep. Bars indicate sequence conservation at every position. (PDF 282 kb) [file 12863_2017_593_MOESM3_ESM.pdf]

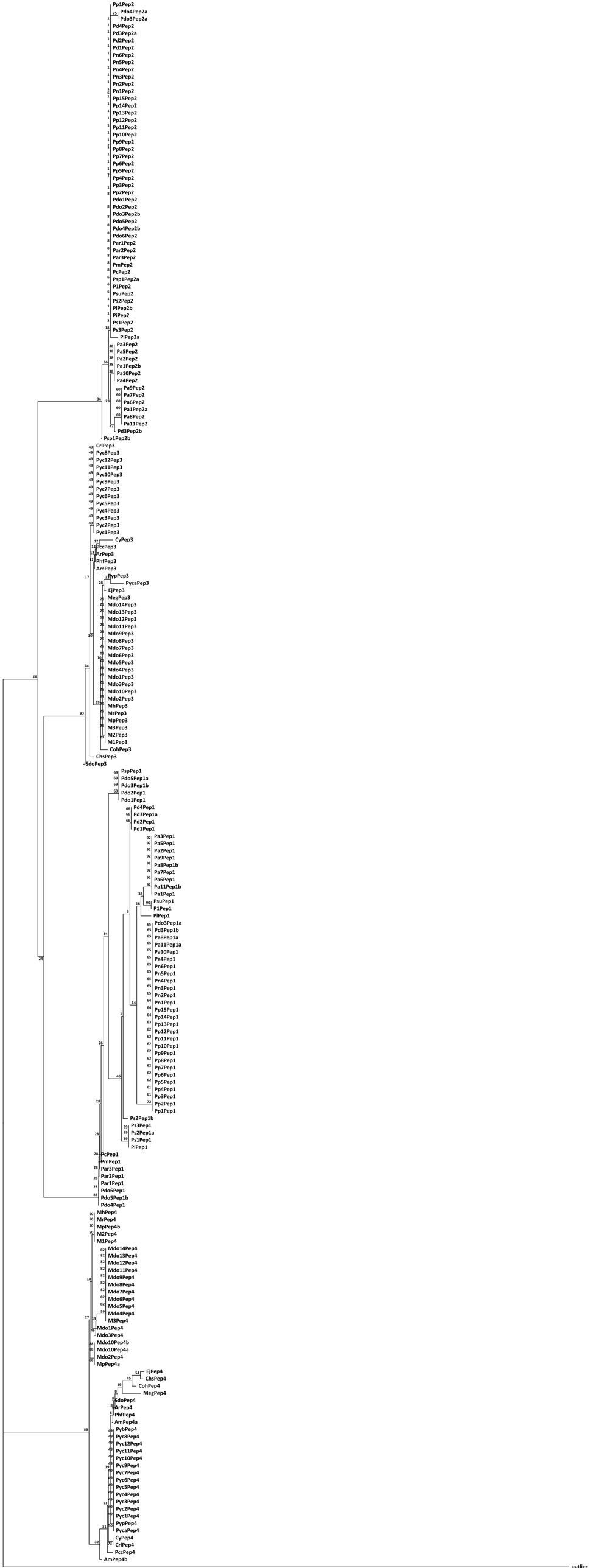

outlier

1,200

Supplement: Supplementary file 5 — Bootstrapped neighbor-joining tree of 214 Rosaceae plant elicitor peptide (Pep) amino acid sequences, constructed using the CLC tool. (PDF 1227 kb) [file 12863_2017_593_MOESM5_ESM.pdf]

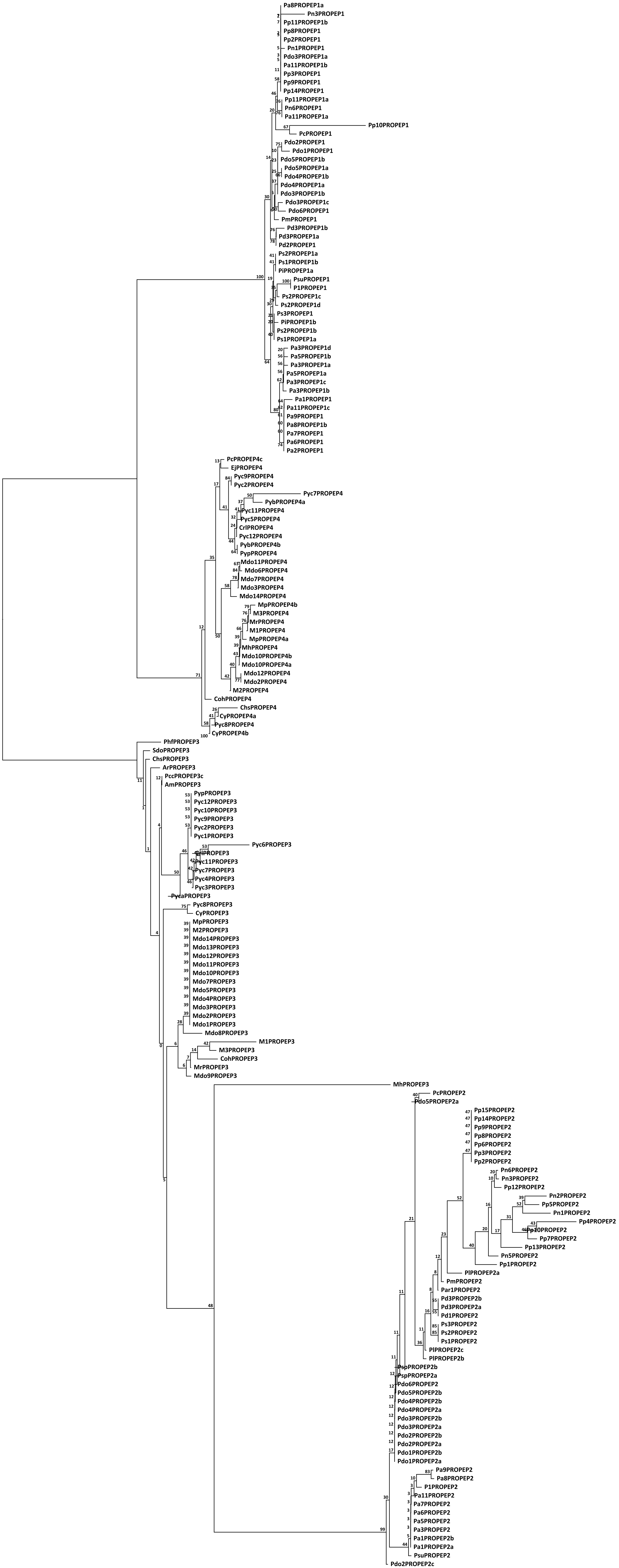

0,500

Supplement: Supplementary file 6 — Bootstrapped neighbor-joining tree of 180 N-terminal regions of PROPEP amino acid sequences from 95 Rosaceae varieties, constructed using the CLC tool. (PDF 1327 kb) [file 12863_2017_593_MOESM6_ESM.pdf]
